# “What would you recommend doctor?”—Discourse analysis of a moment of dissonance when sharing decisions in clinical consultations

**DOI:** 10.1111/hex.12881

**Published:** 2019-03-27

**Authors:** Rebecca Sherlock, Fiona Wood, Natalie Joseph‐Williams, Denitza Williams, Joanna Hyam, Helen Sweetland, Helen McGarrigle, Adrian Edwards

**Affiliations:** ^1^ Division of Population Medicine, School of Medicine Cardiff University Cardiff UK; ^2^ Cardiff and the Vale University Health Board Cardiff UK

**Keywords:** breast cancer, communication, discourse analysis, patient participation, qualitative research, shared decision making

## Abstract

**Background:**

Proven benefits of Shared Decision Making (SDM) include improved patient knowledge, involvement and confidence in making decisions. Although widely advocated in policy, SDM is still not widely implemented in practice. A common patient‐reported barrier is feeling that “doctor knows best”; thus, patients often defer decisions to the clinician.

**Objective:**

To examine the nature of the discourse when patients ask clinicians for a treatment recommendation during consultations when treatment decisions are being shared and to examine clinicians’ strategies used in response.

**Design, Setting and Participants:**

Theme‐orientated discourse analysis was performed on eight audio‐recordings of breast cancer diagnostic consultations in which patients or their partners attempted to defer treatment decisions to the clinician. Clinicians were trained in SDM.

**Results:**

Tension was evident in a number of consultations when treatment recommendations were requested. Clinicians responded to recommendation requests by explaining why the decision was being shared (personal nature of the decision, individual preferences and equivalent survival outcomes of treatment options). There was only one instance where a clinician gave a treatment recommendation.

**Discussion and Conclusions:**

Strategies for clinicians to facilitate SDM when patients seem to defer decisional responsibility include being clear about why the decision is being shared, acknowledging that this is difficult and making patients feel supported. When patients seek guidance, clinicians can provide a recommendation if grounded in an understanding of the patient's values.

## 1 BACKGROUND

A key element of person‐centred care is shared decision making (SDM), in which patients and clinicians work together to make informed treatment decisions by integrating evidence and patient preferences.^1^, ^2^ There is evidence that SDM improves patient knowledge, involvement and confidence in making decisions,^3^ as well as adherence to recommended care.^4^

Although SDM is now widely advocated in policy,^5^, ^6^ it is still not routinely implemented in practice. Studies have highlighted many system, clinician and patient barriers to its routine use.^7^, ^8^, ^9^, ^10^, ^11^ System barriers include time pressures, concern about disruption to established workflows and lack of incentives rewarding SDM.^8^ Clinicians may be unaware of SDM, sceptical about its value, or lack the confidence and skills to incorporate SDM into their routine practice. Clinicians have also expressed concern that discussing all treatment options may lead to inappropriate patient demand; thus, the options offered may be limited by clinicians’ own preferences.^12^, ^13^

Patients may be anxious about engaging in SDM and reluctant to express their preferences for fear of being labelled as difficult or demanding, even when they are well informed.^14^ A commonly cited clinician‐perceived barrier is patient unwillingness to be involved in decision making.^9^ However, a recent systematic review found that many patients currently feel that they *cannot* participate, rather than not *wanting* to.^15^ Patients may undervalue their ability to understand the information given to them, deferring the decision to the clinician, who is “expert.”^15^ Patients may also fear being abandoned to make a decision alone and react by indicating that they do not want to participate in SDM, using phrases such as “please tell me what to do” or “what do you (as a clinician) recommend?”.^15^ This creates dissonance in the consultation; the clinician wants the patient to engage with decision making, but the patient lacks the confidence to take on this responsibility. Clinicians may perceive the patient's questions as unwillingness to engage with SDM and respond by shifting to a more paternalistic approach. Thus, the opportunity for SDM may be closed prematurely.

Studies of how clinicians talk to patients during consultations in which treatment decisions are being made have been undertaken in a number of settings. These include studies of SDM in primary care during discussions of antibiotic expectations,^16^ management of cholesterol,^17^ and in secondary care settings when interpreters are present.^18^ In addition, a study by Robertson et al^19^ has exemplified the complex conversational processes at work between doctors and their patients when sharing decisions in consultations. Their work identified that time constrained clinicians tend to use “partnership talk” to counter resistance and invite consensus. We aimed to build on this by examining the nature of the discourse specifically when patients (or their companions) ask clinicians for a treatment recommendation and examine the strategies used by clinicians to enable SDM to continue.

## 2 METHOD

We used theme‐orientated discourse analysis to examine in detail these moments of dissonance between patients, or/and their companions, and clinicians in the consultation. Discourse analysis provides a framework for systematically analysing face‐to‐face talk.^20^ It focuses on interaction, investigating meaning behind the language that people use, and is therefore well suited to studying the complexities of communication within the clinical encounter.^21^ Discourse analysis has been used in many ways perhaps as a consequence of its multidisciplinary origins and, like many other qualitative methods, there is no unified approach. We used Theme‐Orientated Discourse Analysis as outlined by Roberts and Sarangi.^22^ This approach examines analytical themes such as contextualization cues (intonation, stress and pauses), facework, identity work and rhetorical devices (contrasts, repetitions, metaphors etc). We also analysed linguistic features of the discourse such as the use of laughter and intakes of breath which are more typically associated with analysis of the procedural nature of conversational interactions. Theme‐orientated discourse analysis also examines focal themes within the data.

Data were derived from the Making Good decisions In Collaboration (MAGIC) programme, commissioned by The Health Foundation (UK) to explore how SDM can be embedded into primary and secondary care settings.^9^ Patients with early‐stage breast cancer were recruited consecutively from the breast care centre between April 2014 and September 2015. Patients who were unable to communicate in English or who were deemed unsuitable by the clinical team due to health reasons were excluded. All patients were provided with a study pack before their consultation, which included a cover letter, patient information sheet and consent form. Patients were offered the decision between mastectomy or lumpectomy (wide local excision) with follow‐up radiotherapy.

Two consultations were audio‐recorded for each of the 25 patients (50 consultations in total)—their initial diagnostic consultation at the Breast Cancer Centre and the follow‐up visit one week later. Family members or companions were sometimes present and their interactions have also been included in the analysis as the contribution of companion interactions within the consultation are of importance.^18^, ^23^ The breast care team had been part of a SDM implementation programme, contributing to the development of SDM interventions and tools. All of the clinicians had received workshop training in SDM following the 3‐talk model of SDM,^24^ which describes three key steps to SDM, namely: choice talk (ensuring the patient knows that a choice is available and their input is important), option talk (providing detailed information about options) and decision talk (supporting the patient to consider preferences and deciding what is best). Consultations were audio‐recorded with written informed consent from patients and clinicians.

**Figure 1:**
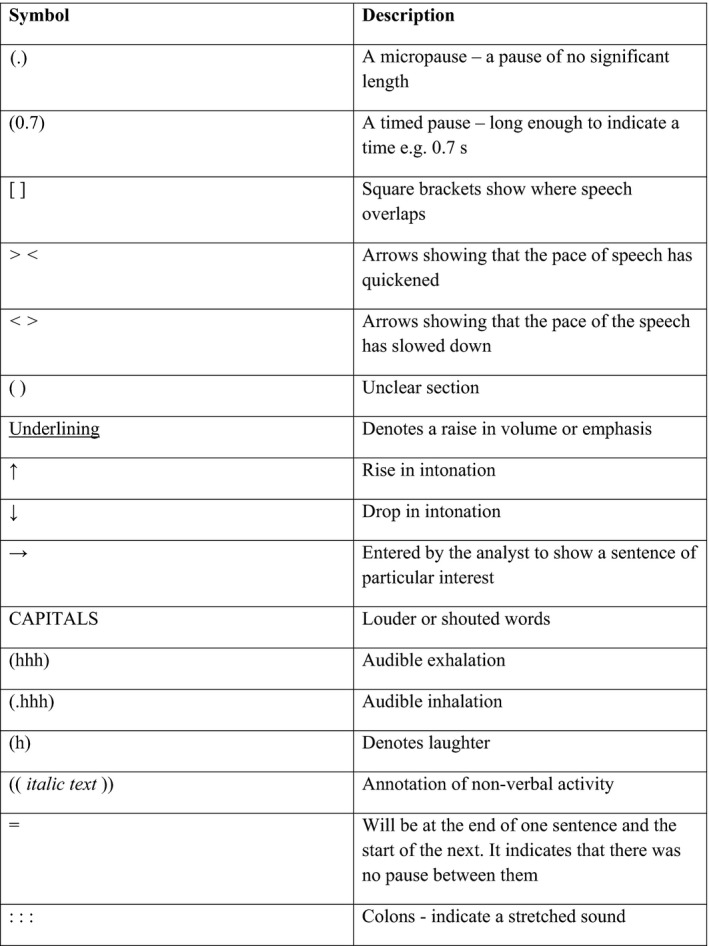
Transcription notation

We searched all 50 consultations for instances when a treatment recommendation was sought. All data were transcribed, but talk preceding or following these requests were transcribed in more detail (see Figure 1). One  research (RS) listened to the audio‐recordings multiple times while reading the transcripts. Other team members (FW and JH) independently read and commented on all of the transcripts. Analytical reflections were guided by considerations of how the discourse made the analyst feel (for example, amused, uncomfortable) and what was present or missing from the discourse (for example, an apology, pauses, emphasis).^25^ Emerging ideas were discussed among the research team at fortnightly meetings throughout the analysis phase and refined accordingly. Although some of the clinicians were female, all clinicians are in this paper are referred to as male in order to preserve anonymity and to ensure clarity in the transcripts with patients who were all female.

## 3 RESULTS

In consultations with eight of 25 patients, there were moments when either the patient or their partner attempted to defer treatment decisions to the clinician: six where the patient asked the clinician for a recommendation, and another two cases where the patient's partner asked. One patient indicated on five separate occasions during her two consultations a desire for the clinician to take decisional responsibility.^26^

### 3.1 Structure of the consultations

All of the consultations followed a similar structure. In the diagnostic consultation, the clinicians explained that the biopsy result confirmed early‐stage breast cancer. Clinicians then used the 3‐choice model^24^ introducing the notion that treatment choices were available and that their input to the decision was important. Following this, they explained the two treatment options, that survival outcomes are equal for these, and that the patient can choose which treatment they prefer based on what fits best with their own personal preferences. The patient was then given the opportunity to ask questions; this was generally the point in the consultation when the patient sought a treatment recommendation. Written information, in the form of a brief decision aid comparing the treatment options, was given to each patient to take home and inform their decision.

The second consultation was a home visit about a week later by a specialist nurse. The patient's decision process was discussed, any misconceptions clarified and questions answered. Most patients came to a treatment decision between these two appointments, and all reached a decision by the end of the second consultation.

We noted two main themes that emerged when patients or their partner sought treatment recommendation. These were as follows (a) tension in the consultation when treatment recommendations were sought, and (b) strategies used by clinicians in response to a treatment recommendation. Each of these will be explored in further detail below while attending to the discursive features of the interaction.

### 3.2 Tension in the consultation when treatment recommendations were sought

Patients anticipated the clinician's reluctance to give a treatment recommendation, even before they asked for it. This may have been because the clinicians had already introduced the concept of patient choice and justified why the choice was being offered earlier in the consultation.

In feature 1 below we see patient B's hesitancy in phrasing her question indicated by the micropauses and incomplete utterances suggests that she suspects the clinician will not recommend a treatment option for her. She anticipates the clinician's answer to her own question “or is it up to me?” and her quick reply of “okay” indicates that she was expecting this answer.

Patient B: Umm (.) are you (.) do you recommend diff‐ or is it up to me?

Surgeon: ↓No

Patient B: =No= ((murmours))

Surgeon: it's everything's up to you=

Patient B: =Okay=

In feature 2 patient G's partner recognizes that it is difficult for the clinician to give a recommendation, acknowledging that this is putting the clinician “on the spot.” This is a type of pre‐emptive repair work, where the speaker knowingly introduces tension to the conversation, but undertakes remedial work in advance to lessen the conflict it causes.^27^ Challenging more powerful participants requires softening or mitigating phrases such as the partner's use of “I obviously think” and emphasizes the ritual element and fragility of the relationship.

Partner G: So would you (.) no if (.) if you were put on the spot, or say, what would you (.) uh if I put you on the spot, what would you recommend? Take as much as you can away (.) or as little? I obviously think what you’re telling me is take as much as you can away, do it in one go

Surgeon: N‐no, I mean [that's why]

In feature 3 patient H's partner prequels his question with “can I ask just the one question?” which serves to both emphasize the importance he places on his next question and to make it more difficult for the clinician to decline to answer. Expressing tentativeness, known as “hedging” (seen here by seeking the clinician's permission to ask the question) is a form of linguistic politeness, and a way of the speaker showing that he is in a powerless position, or feels the need to act that way in order to serve his own purpose. It is also another example of pre‐emptive repair work. Such devices are used unconsciously and their function is often to establish or reinforce social relations such as the power imbalance from the doctor and the patient.

Partner H: Can I, is, can I ask just the one just the one question? In this situation

Surgeon: Mmm

Partner H: Generally (.) what do people just go for ‐ the complete removal or just the lump?

The exchange with patient C in feature 4 also shows a moment of dissonance for both patient and clinician which then requires some repair work. The clinician's laughter in his response could indicate the awkwardness this question causes him, as he feels unable to provide an answer. The work of Erving Goffman is useful here in his ideas of the presentation of self and maintaining “face,” that is the positive self‐image one holds when interacting with others. When something happens to damage face, repair typically occurs by one or other of the parties.^27^ Here, this is done by the patient laughing at herself “that was a silly thing to say then, wasn't it” and the clinician's reassurance that no damage has been caused “no no no it's fine.” The patient seems to be apologizing for deviating from the clinician's plan for her to take decisional responsibility. The clinician then tries to repair the damage caused by this moment of conflict in their agendas.

Surgeon: There’s no difference in terms of the outcome (.) so it’s really how you feel about it

Patient C: Well what would you initially recommend then? Put it that way

Surgeon: Well we don’t recommend ((laughs))

Patient C: No okay

Surgeon: that’s why it’s a patient [choice] ((laughing))

Patient C: [choice] okay

Surgeon: Yea (.) because they’re equal

Patient C: [Now] that was a silly thing to say then, wasn’t it?

Surgeon: No no no it’s fine

In feature 5, the clinician explicitly shares the difficulty in answering the partner's request for a treatment recommendation, having never been in the patient's position. The clinician's intake of breath and then utterance of “you know” is mark of a change in interactive frame and shifts the doctor‐patient/companion interaction towards a frame of equals having a conversation. The cues herald a change in footing from an objective, scientific, professional role, to a more personal, emotional one, akin to friend. We also get an insight into the personal tension experienced by the clinician when faced with a request for a treatment recommendation.

Surgeon: I think this would be appropriate for both options=

Partner G: =Yea=

Surgeon: =Both equal long term studies have shown that they both are the same=

Partner G: =Yea=

Surgeon: =and it is completely safe so (.hhh) you know I’ve never been in your wife’s position so it’s very difficult for me to say what I would do=

Partner G: =Mmm=

Whereas most patients in the study seemed to quickly accept SDM, Patient A (feature 6) was very resistant to taking decisional responsibility, attempting to defer the decision on five separate occasions (see Box ). She is explicit about her expectation for the clinician to take decisional responsibility—“you've got to say well I think it's better for you to have this.” Her use of imperative language implies that he has an obligation to do so, as the specialist. Addressing the clinician as a “specialist” serves to reaffirm the social relationship of expert versus non‐expert, indexing her expectations of the relationship.

Patient A: Well (.) I’m going to leave it in your hands=

Surgeon: =Okay=

Patient A: =And you decide which is best for me, whether it’s a (.) a lump::ectomy or a mast::ectomy, whatever

Surgeon: Okay (.) <Unfortunately I can’t decide for you>

Patient A: [Bu‐]

Surgeon: [Uh]

Patient A: But you’re the specialist

Surgeon: ↑I know! [But]

Patient A: [But] you’ve got to say

Surgeon: [Aa]

Patient A: Well I think it’s better for you to have this

### 3.3 Strategies used by clinicians in response to patients’ requests for a recommendation?

A number of different strategies were used by clinicians in response to patients’ treatment recommendation requests.

We will explore these types of responses in further detail below, and recurrent features of the discourse around these.

#### 3.3.1 Restating the importance of individual preferences

One strategy was the justification of patient choice by acknowledging individual priorities and preferences. The selective use of personal pronouns appeared to be of relevance. Sometimes clinicians used the third person to discuss treatment choice in a generalized way:

"People, people vary as to how they think they'll deal with a mastectomy"

(Patient H) Sometimes the more direct second person was used, emphasizing that the choice is personal to that individual patient:

"So really it's < what is important for you > what are the factors that *you* will think after reading this material or having a look at this options grid < what will be important for *you*>" (Patient B)

There was also evidence of clinicians “normalizing” SDM when they were justifying patient choice:

"But we do realize that it’s important that our patients get that choice."

The use of the collective pronoun “we” and “our” helps to authenticate this assertion, showing the patient that other clinicians also support this approach, and it is normal practice. This may make it more difficult for a patient to raise their concerns about participating in SDM, as it is implied that it is routine for patients to make their own choices.

#### 3.3.2 Clinicians’ use of emotional language

Some clinicians discussed the decision in emotional terms, placing importance on how the patient feels about it, and the aim for them to feel in control and content with their decision.

In the exchange with patient C an H below, the clinician repeatedly and emphatically use emotional words such as “feel” and “content,” demonstrating the value that they place on emotion‐focused coping,^28^ and using this to rationalize the patient deciding on their own treatment. Their use of language aims to empower the patient “it's important too that you feel in control,” and acknowledges the importance of the patient feeling positive about their decision.

Surgeon: Well‐ that’s why it’s your choice you see because (.) it’s how you feel about it

Patient C: Right okay

Surgeon: It’s not how (.) we feel about it (.) and that’s why we must

Patient C: [w‐]

Surgeon: [say to you] there’s no difference in terms of the outcome (.) so it’s really how you feel about it

*Patient H*

Surgeon: Umm so that’s why it’s, you know, we give people the choice because it’s important too that you feel in control, it’s not me saying this is the way you have to have it done

Patient H: Mmm

Surgeon: Because I think everybody, and I think that’s what we (.) appreciate the fact that everybody is different

Patient H: Mmm

Surgeon: The important thing is that you are content with the decision that you make

#### 3.3.3 Emphasizing that treatment options have equal outcomes

When asked for their recommendation, some clinicians responded that either option would be appropriate. Some clinicians responded to the patient's recommendation request with phrases such as “I think any of those options are (.) very appropriate”; “I think this would be appropriate for both options=.” This was justified with the equality in treatment survival outcomes. By validating all treatment options, the clinicians are perhaps reassuring patients that they cannot make a “wrong” decision in terms of survival outcomes. This is in contrast to the potential for a “wrong” decision in terms of personal outcomes such as quality of life, psychological and emotional impact of treatment.

#### 3.3.4 Language indicating decisional responsibility

In the exchange with patient H (above) the clinician's use of “we” and “you” clearly divides the patient from the clinical team. This language seems to contradict the idea of decision making being shared and the discourse in this example focusses more on the actual decisional responsibility (who makes the decision) rather than the process of involvement (exchange of information and exploring preferences for who makes the decision). Pronouns can be used to include or exclude groups, or indeed obscure the identity of the group because it is not clear whether the clinician is referring to “we” as the immediate clinical team or health professionals more generally. For some patients who are prepared to make their decision independently, the language indicating ownership of “*your* choice” could be empowering. However, for those patients who are less confident in taking decisional responsibility, this language risks instilling feelings of abandonment. Where decisional responsibility becomes mandatory it ceases to promote patient empowerment.^29^

In fact throughout all of the studied consultations, clinicians used language which indicated that the choice was the patient's alone to make. For example:

Clinician: And then you can decide and tell us that (.) I'm (.) I want to have a mastectomy, or I would prefer to have a wide local excision (Patient B)
Clinician: It's just for you to decide (Patient E)

### 3.4 Clinicians giving a recommendation

There was one occasion when the clinician gave a recommendation when asked (see patient E).

Patient E: And (.) what would your recommendation (.) be at this point?

Surgeon: Urr, well, I can just give you information, and just just thinking about the size of it as well that’s what I’m saying, I think that you ((clears throat)) cause its quite small lesion I think it’s it’s, what I would be thinking of (.) uhh considering all of the information that you will have (.) uhh I would think that this is perfectly suitable for the just removing the, the, the tissue (.) and checking the lymph nodes [I wouldn’t go for]

Patient E: [So what happens if the lymph nodes are affected?]

Surgeon: What happens if the lymph nodes are affected? If they are affected (.)

((Then later on in the consultation)).

Surgeon: Different things are important to uh to everyone

Patient E: uhhmm

Surgeon: So think about it

Patient E: uhhmm

Surgeon: As I said (.) the both things can be done, I (.) think that you are very well suited for the, going forward, for the small procedure, just removing part of the breast only (.) but you will go through the grid and you will decide for yourself

Patient: uhhmm okay

The clinician initially implies that they are not allowed to give a recommendation “urr, well, I can just give you information,” before tentatively offering a recommendation. The clinician's hesitant language with pauses, throat clearing and “hedges” (eg, “I think it's, it's” “uhh considering all of the information that you will have”) could indicate their discomfort in answering the patient's question. The clinician emphasizes it is their personal opinion “what *I* would be thinking of,” and later reiterates that it is the patient's choice.

## 4 DISCUSSION

### 4.1 Summary of main findings

Of 25 patients in our data set, only eight patients or their partners sought a treatment recommendation. Our discourse analysis focused on these eight patients in order to study how patients made these requests and how clinicians handled them. Thus, most (17) patients did not ask the clinician for a recommendation at any point during their consultations.

Our analysis highlights the difficulty for both doctors and patients in achieving SDM, particularly when patients express reluctance or anxiety about participating in the decision‐making process. We have identified a number of features within the procedural nature of the conversational interaction including repair work, micropauses, incomplete utterances, and laughter. It appears to be within these conversational features when moments of dissonance occur. There was evidence of tension being experienced by both parties in the consultation when a treatment recommendation was sought and that for some patients there remained the feeling that “doctors know best.” That said, clinicians employed strategies to enable SDM to continue. One strategy used by clinicians was to explain why the patient was being given a choice; namely the personal nature of the decision based on individual preferences, priorities and emotional reactions, as well as the equal survival outcomes of each option and thus no possibility of making a “wrong” decision. Clinicians also reassured the patient they would have sufficient time to make a decision. One clinician gave a treatment recommendation, while also emphasizing the importance of patient preferences. We were also surprised at the lack of “meta‐communication” (talk about talk) used by the clinical team in response to patients’ treatment requests. For example, the requests might have usefully triggered meta‐communication for the clinicians to return to discussing the rationale for a decision process itself.

### 4.2 Comparison with other literature

#### 4.2.1 SDM is complex and challenging

It is widely acknowledged that SDM is challenging to achieve in everyday clinical practice.^2^, ^30^ The clinicians in our study were trained and committed to SDM; yet, they and their patients still encountered tension in their consultations. This is unsurprising given the complexities of sharing decisions, particularly in the emotionally charged context of cancer treatment when decisions have major consequences for patients and their families. Both patients and clinicians undertook remedial work during the consultations to repair these moments of tension.^27^ That said, our study corroborates previous research findings^31^ that, with the right communication attitudes, skills and tools, clinicians can enable SDM conversations to continue, even when patients seek a treatment recommendation.

#### 4.2.2 Should clinicians give a treatment recommendation if asked by patients?

This study raises an important question: Should clinicians give a recommendation if asked by patients? In our analysis, we sensed a reluctance among the clinicians to provide a treatment recommendation. There was only one instance where a clinician gave a recommendation when asked, and this was given hesitantly and quickly qualified with the importance of patient preference.

Being asked “what would you recommend doctor?” can understandably make clinicians feel uneasy, especially in the context of “trying to do SDM.” Providing a recommendation risks threatening patient autonomy, while declining to answer may leave patients feeling abandoned.^32^ Emmanuel and Emanuel's “interpretative model” of the physician‐patient relationship advocates a compromise between these, where the clinician guides the patient in identifying their personal values and the medical interventions that go along with those values.^33^ So when patients seek guidance, clinicians should ground their advice in not just a medical diagnosis, but also an understanding of the patient's values and priorities.^34^ Such involvement should not be seen as an infringement on autonomy, but rather as a way of respecting it. Addressing this in SDM clinician training should empower clinicians to respond to patient recommendation requests effectively.

#### 4.2.3 Use of language indicating decisional responsibility

Although the analysed consultations did demonstrate SDM, this was not necessarily reflected in the clinician's use of terminology with patients. Their language indicated patient ownership over their decision, implying that ultimately the patients would make the decision themselves. This fits with the “informed choice” model of decision making: the clinician's role is to provide the patient with sufficient information for them to make an informed choice.^2^ However, it has been suggested that patients need more than accurate information: they need to feel supported, accompanied and cared for. Inclusive language such as “we,” “us” and “together” could make patients feel more supported in the decision‐making process and should be incorporated into SDM training.

### 4.3 Study strengths and limitations

This study analysed real‐life consultations, allowing exploration of practical issues at the coal‐face of clinical practice. Our conclusions should therefore be relevant to practicing clinicians striving to achieve SDM with their patients.

Our study was a theme‐orientated discourse analysis of consultations with eight patients led by clinicians trained in shared decision‐making skills, so our findings are not representative of all clinicians’ skills or all patient scenarios. The detailed nature of discourse analysis necessitated that only sections of consultations were analysed. This led to drawing artificial boundaries in the transcripts, potentially disrupting flow and context.

The consultations were audio‐recorded so we were unable to analyse non‐verbal communication, an important aspect of any face‐to‐face interaction. It is also possible that the presence of a recording device in the room may have affected communication processes, and clinicians may have been more careful to avoid giving a treatment recommendation if they perceived this to be “disallowed” in SDM.

### 4.4 Implications for practice

We recommend a number of strategies that could be emphasized and, where lacking, built into SDM training in order to help clinicians deal with situations in which patients seek a treatment recommendation:

Explain **why** the patient is being involved in decision making
**Acknowledge** that it can be difficult to make a decision
Give the patient **time** to make the decision where possible, and reassure of this
Make the patient **feel supported** in the decision‐making process by using language which implies a team approach, such as “*we* can decide *together*”
Explore the patients’ **preferences and priorities**, and help them identify which treatment options best fit those values
When patients seek guidance, clinicians **can** provide a treatment recommendation, as long as (a) the patient understands that their input in the process is valued, (b) the pros and cons of all options have been discussed in detail and understood by the patient, and (c) the patient's views, concerns and preferences have been sufficiently addressed.

## 5 CONCLUSION

We conclude that patients should be supported to remain involved in SDM even if they seek a treatment recommendation. We suggest several strategies for clinicians when responding to such patient requests. Most importantly, clinicians can provide a treatment recommendation when patients seek guidance, as long as it is grounded in an understanding of the patient's values and preferences.

## CONFLICT OF INTERESTS

The authors have no conflict of interests to declare.
